# Worse and Worse and Worse: Essential Tremor Patients’ Longitudinal Perspectives on Their Condition

**DOI:** 10.3389/fneur.2016.00175

**Published:** 2016-10-13

**Authors:** Jesús Gutierrez, Jemin Park, Olufunmilayo Badejo, Elan D. Louis

**Affiliations:** ^1^Division of Movement Disorders, Department of Neurology, Yale School of Medicine, Yale University, New Haven, CT, USA; ^2^Department of Chronic Disease Epidemiology, Yale School of Public Health, Yale University, New Haven, CT, USA; ^3^Center for Neuroepidemiology and Clinical Neurological Research, Yale School of Medicine, Yale University, New Haven, CT, USA

**Keywords:** essential tremor, clinical, epidemiology, prognosis, longitudinal

## Abstract

**Background:**

Essential tremor (ET) patients regularly inquire about their prognosis. Therefore, physicians have cause to review available medical literature for meaningful answers. Longitudinal studies are ideally suited to provide a glimpse into the evolution of tremor. Despite its high prevalence, there are surprisingly few longitudinal clinical studies of ET. Furthermore, none of them provide data from the patients’ perspective. Understanding the patient vantage point is valuable as it is the starting point of personalized medicine. Given the progressive nature of ET, we hypothesized that many patients will experience an increase in symptom severity over time. However, due to a lack of clinical data, the exact nature of this progression is unclear. For example, whether patients experience a worsening at each time interval is simply not known. In this longitudinal study, we assessed whether ET patients felt that their symptoms had worsened between each follow-up evaluation and try to identify specific clinical characteristics associated with this experience.

**Methods:**

A cohort of 164 ET cases enrolled in a prospective, longitudinal research study. After a baseline in-person assessment, they received regular telephone evaluations for up to 5.25 years, beginning in 2009. During each follow-up evaluation, cases answered the question, “has your ET worsened since our last call?”

**Results:**

Two-thirds [104 (63.4%)] of ET cases reported worsening at one-half or more of their follow-up evaluations. Furthermore, one in four cases [44 (26.8%)] reported worsening at every follow-up evaluation. Self-reported worsening was not associated with any of the baseline clinical variables assessed, including age, gender, tremor duration, age at tremor onset, or total tremor score.

**Conclusion:**

Little has been written from the patients’ perspective on progression of ET. When followed longitudinally at regular intervals, a majority of ET cases we studied reported worsening one-half or more of the time; furthermore, one in four cases reported worsening at each and every assessment, indicating that they felt they were inexorably getting worse and worse with time. That there is so much self-reported worsening in ET argues against the notion that this is a static and benign condition. It suggests that patients experience it as a condition that worsens regularly and consistently.

## Introduction

Essential tremor (ET) is one of the most prevalent movement disorders and is commonly encountered in clinical practice (1–3). As it is a chronic and progressive disorder, patients regularly inquire about their prognosis. Hence, physicians have cause to review available medical literature for meaningful answers. Longitudinal studies are ideally suited to provide a glimpse into the evolution of tremor in ET patients. Despite this, there are surprisingly few longitudinal clinical studies of ET. Indeed, there are five longitudinal studies (4–8), although one was a retrospective review of clinical records (4). Four of these studies reported cohorts of modest size (<50 cases) (4–6, 8). One of these studies focused on baseline prognostic factors to predict arm tremor severity over time (4); another examined the change in tremor frequency using accelerometry and electromyography (5). Only one study evaluated the change in arm tremor severity using a standardized clinical rating scale (6). The two other studies did not provide any data on change in severity of arm tremor over time (7, 8). Most germane to the current analyses is that none provided data from the patients’ perspective.

In actuality, there is remarkably little written about either the experience of tremor or the experience of the evolution of tremor from the perspective of the ET patient. Although a patient-centered approach is less objective than the one provided by clinician-assigned ratings or tremor analysis, understanding the patient vantage point is valuable as it is a central ingredient as well as the starting point of personalized medicine (9).

Given the progressive nature of this disorder, we hypothesize that many patients will experience an increase in symptom severity over time. However, due to a lack of clinical data, the exact nature of this progression is not clear. Whether patients experience a worsening at each time interval is simply not known. As part of an ongoing clinical research study, we prospectively collected longitudinal data at regular intervals on *self-reported* symptom progression. At each interval, 164 cases were asked by telephone “has your ET worsened since our last call?” The goal of this report was to (1) assess whether ET patients feel their symptoms worsen at each interval and (2) identify the specific clinical characteristics that may be associated with the experience of worsening.

## Materials and Methods

### Case Ascertainment

Essential tremor cases were recruited through the essential tremor centralized brain repository (ETCBR), which serves as a centralized repository for the prospective collection and study of brains of ET cases throughout the United States (10). ET cases were recruited as future brain donors through advertisements on patient advocacy group websites and the ETCBR website (www.essentialtremor.us). Each recruited case underwent an in-person baseline evaluation. This was followed by regular follow-up telephone assessments, the purpose of which was to update clinical information. Enrollment of the wave of cases reported here began in March of 2009 and ended in April of 2011. Follow-up has continued to present.

### Baseline Evaluation

Once enrolled, ET cases were visited in their homes by a trained research assistant, who performed a detailed baseline evaluation. Each case signed an informed consent form approved by the Internal Review Board of Columbia University. The research assistant administered a series of semistructured clinical questionnaires eliciting demographic and medical information. Medical comorbidity was evaluated using the Cumulative Illness Rating Scale (CIRS, range 0–42) (11). To briefly assess cognition, the Folstein Mini-Mental State Examination (MMSE, range 0–30) was administered (12). Additionally, each case drew four standardized, hand-drawn Archimedes spirals (two right, two left), each on an 8.5″ × 11″ sheet of paper.

The research assistant obtained a standardized, videotaped neurological examination, including a detailed assessment of tremor (13). The videotaped examination included assessments of postural tremor (two positions), kinetic tremor (five activities with each arm), and intention tremor of the arms, as well as head tremor. Tremor was rated by a senior movement disorder neurologist (Elan D. Louis) using a reliable (14) and valid (15) clinical rating scale, which included ratings from 0 to 3 for each of 12 items. A total tremor score (range 0–36) (6), which is a measure of action tremor, was calculated based on these ratings. The videotaped examination also included the motor portion of the Unified Parkinson’s Disease Rating Scale (16).

### ET Diagnoses

Essential tremor diagnoses were carefully assigned and then confirmed using the following methods. First, the vast majority of cases were diagnosed clinically with ET by their treating physician (either by their primary health physicians or neurologists); the remaining few were self-diagnosed cases and included individuals with a strong family history of ET. Second, as noted above, cases were asked to complete a series of semistructured clinical questionnaires (demographic data, general medical data including medications, and tremor-specific data), which included data on age of onset and family history information. As noted above, Archimedes spirals were also drawn. These data were supplemented with additional clinical information (from clinical records, treating physicians, and family members). ET diagnoses were then confirmed by a senior neurologist specializing in movement disorders (Elan D. Louis) who used the following criteria: (1) moderate or greater amplitude arm tremor (rating ≥2) in at least one of the submitted Archimedes spirals; (2) no history of Parkinson’s disease or dystonia; and (3) no other etiology for tremor (e.g., medications, hyperthyroidism). Third, as noted above, ET cases underwent a standardized, videotaped neurological examination. Each videotape was reviewed (Elan D. Louis), and based on the questionnaire and videotape data, the diagnosis of ET was re-examined in each case using published diagnostic criteria (moderate or greater amplitude kinetic tremor (tremor rating ≥2) during three or more videotaped activities or a head tremor in the absence of Parkinson’s disease or other known causes) (14, 15, 17).

### Follow-up Evaluations

Cases received regular follow-up evaluations by telephone. The protocol was approved by the Internal Review Boards of Columbia and Yale Universities. During the initial 3 years of the study, these evaluations were performed at 6-month intervals. After that point, the interval was lengthened to 9 months due to concerns about participant fatigue.

During each follow-up evaluation, the research assistant assessed progression of symptoms. Thus, cases answered the question, “has your ET worsened since our last call?” If cases answered “Yes” to this question, the research assistant asked them, “how has your ET worsened?” Cases were also asked about changes in medications and ethanol consumption, and they submitted four new standardized Archimedes spirals (13). Finally, they were asked a series of screening questions for Parkinson’s disease and dystonia. If any screening question was positive for Parkinson’s disease or dystonia or if a spiral showed signs of micrographia, the research assistant revisited the case at home to obtain a follow-up videotaped neurological examination.

### Additional Evaluations

Medications were recorded at baseline and each follow-up telephone interview. In order to consider the potential effects of medication changes on the reported change in tremor symptoms, a “medication change score” was created. The medication list of each case was reviewed to identify tremorogenic and tremor-reducing medications. A medication was deemed tremorogenic based on previously published reviews (18) and included lithium, β-adrenergic agonists, thyroxine, and other agents. Similarly, a medication was labeled as tremor reducing based on published data (19). Each tremorogenic medication was assigned a value of 1; also, each tremor-reducing medication, a value of −1. A baseline score was calculated as the sum of all these values at baseline, and a follow-up score was calculated as the sum of all these values at the most recent follow-up evaluation. The final “medication change score” was calculated as the difference between the baseline medication score and the most recent follow-up medication score; a negative value indicated greater use of tremor-reducing medication at follow-up.

Similarly, to consider potential effects of changes in daily ethanol intake on reported change in tremor symptoms, a “change in ethanol intake score” was created. This was based on the average number of drinks per week that each case reported consuming at each evaluation. The change in ethanol intake score was calculated as the change in number of drinks per week reported between baseline and the most recent follow-up.

Finally, to obtain a more objective measure of change in tremor severity with time, two trained research assistants (Jesús Gutierrez and Jemin Park) rated all of the baseline and most recent follow-up spirals using the Bain and Findley 10-point scale (0 = no detectable tremor to 9 = severe tremor) (20). This rating scale has been validated in previous studies assessing tremor severity (21–23). The two research assistants were trained by independently rating 50 Archimedes spirals, and their agreement with those of the senior movement disorders neurologist was substantial (24) [weighted kappa (κ) = 0.63 and 0.64]. To assess the change of spiral ratings over time, a “change in total spiral score” was calculated. Each case submitted two right hand spirals and two left hand spirals. The 0–9 ratings of each spiral were averaged. The change in total spiral score was calculated as the difference between the baseline and the most recent follow-up scores.

### Final Case Selection

Of the 177 cases who had a baseline evaluation, 13 (7.3%) were excluded due to lack of follow-up data (5 deaths, 1 moved outside of the United States, 4 withdrew due to loss of interest, 1 co-diagnosed with dystonia, and 2 miscellaneous). The final sample for analysis (164 ET cases) was similar to the 13 excluded cases in terms of baseline total tremor score (23.8 ± 6.2 vs. 22.6 ± 9.4, *p* = 0.56) and age at onset of tremor (42.6 ± −23.0 vs. 38.4 ± 20.7 years, *p* = 0.60). They did not differ by education (15.0 ± 3.2 vs. 13.4 ± 3.7 years, *p* = 0.18) or gender [62 (37.8%) women vs. 7 (53.8%) women, *p* = 0.25]. The two groups differed by age (83.3 ± 5.6 vs. 88.7 ± 6.3 years, *p* < 0.01).

### Statistical Analyses

Statistical analyses were performed using SPSS (version 21.0; Chicago, IL, USA). The main variable of interest, self-reported worsening was not normally distributed (Kolmogorov–Smirnov test = 0.13, *p* < 0.01). Therefore, to explore the demographic and clinical correlates of the cases’ self-reported worsening, we used Spearman’s correlation coefficients and the Mann–Whitney test. Subsequently, we distributed cases into quartiles based on the percentage of time that they reported worsening. We compared quartiles by demographic and clinical variables using chi-square tests and one-way analysis of variance (ANOVA) when variables were normally distributed. For measures that were not normally distributed, non-parametric approaches were used (e.g., Kruskal–Wallis test). The Jonckheere–Terpstra test was used when we tested hypotheses that included *a priori* ordering.

When we explored the correlation between percentage of times cases reported worsening and change in total spiral scores, we also stratified our analysis by whether or not cases were taking tremor-reducing medications.

## Results

### Case Characteristics

The mean baseline age of ET cases was 83.3 years, and the mean age at tremor onset was 42.6 years (Table 1). On average, ET cases completed 6.8 follow-up assessments (median = 7, range = 1–10) spanning an average time interval of 3.7 years (median = 4.3, range = 0.5–5.25 years).

**Table 1:** **Baseline demographic and clinical features of 164 ET cases**.

Baseline age (years)	83.3 ± 5.6 [84]
Female gender	102 (62.2)
Education (years)	15.0 ± 3.2 [16]
Family history of ET	42 (25.6)
Tremor duration (years)	40.7 ± 22.5 [35.5]
Age at tremor onset (years)	42.6 ± 23.0 [45]
Total tremor score	23.8 ± 6.2 [23.5]

*Data are mean ± SD [median] or number (percentage)*.

### Self-Reported Worsening

**Figure 1:**
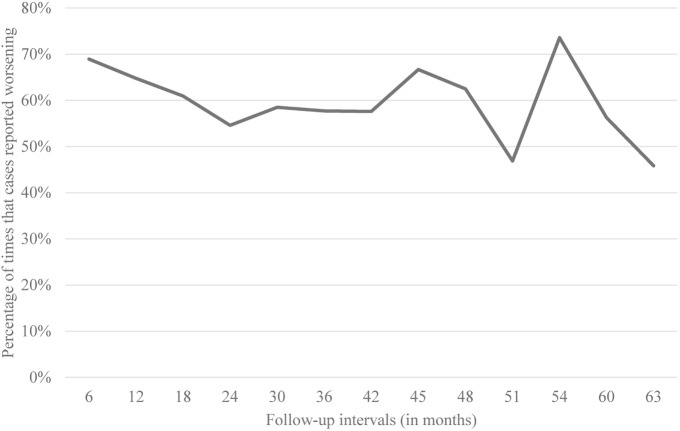
**Percentage of times that cases reported worsening per follow-up interval**.

Of the 164 cases, 44 (26.8%) reported worsening of symptoms at every follow-up interval, 104 (63.4%) reported worsening during one-half or more of the follow-up intervals, and 145 (88.4%) reported worsening at one or more follow-up intervals. Only 19 (11.6%) never reported worsening. Data are shown (Figure 1). We obtained similar results when we restricted our analysis to 32 cases who were never on any medications for the treatment of tremor.

When cases reported worsening of symptoms, they were asked “how has your ET worsened?” Their open-ended responses were collapsed into the following basic categories (Table 2): I am shaking more (34.0%), certain activities are harder to accomplish (33.1%), my medicine is not working as well (6.0%), balance issues (5.9%), my tremor is harder to control (3.3%), and other/miscellaneous (17.7%).

**Table 2:** **Responses to follow-up question, “how has your essential tremor worsened?”**.

Response category	Sample quote from cases	Number (%) of responses in each category (data summed from all follow-up intervals)
I am shaking more	“I am shaking more in every way”	261 (34.0)
	“Shaking more in hands and legs”	
	“Voice is more tremulous, as are hands”	
Certain activities are harder to accomplish	“Writing is impossible; eating is a real problem”	254 (33.1)
	“Hard to write with one hand; no sense of taste”	
My medicine is not working as well	“Propranolol doesn’t help as much as it used to”	46 (6.0)
	“Nadolol is no longer effective”	
Balance issues	“Balance is shot; walking is difficult”	45 (5.9)
	“Badly affected balance; walking unsteady”	
My tremor is harder to control	“Shaking is harder to control”	25 (3.3)
	“Used to be controlled better than it is now”	
Other/miscellaneous	“No way of answering: I’ve changed meds so much I can’t tell which is which”	136 (17.7)
	“Only when agitated”	
	“Can’t judge”	
	“Some days worse than others”	

### Clinical Correlates of Self-Reported Worsening

To assess the clinical correlates of self-reported worsening, we performed several analyses. First, we assessed the correlation between percentage of times that each case reported worsening and a range of demographic and clinical variables (Table 3). There was no association between worsening of symptoms and baseline age or gender (Table 3). There was a marginally significant negative correlation between years of education and reported worsening (Spearman’s rho = −0.14, *p* = 0.08). Similarly, medication change score and reported worsening were marginally correlated (Spearman’s rho = −0.12, *p* = 0.12). There was also a marginally significant positive correlation between CIRS scores and reported worsening (Spearman’s rho = 0.12, *p* = 0.12) and MMSE scores and reported worsening (Spearman’s rho = 0.13, *p* = 0.10). There was no correlation between reported worsening and any of the other clinical features (e.g., tremor duration, age at tremor onset, and total tremor score, Table 3). Finally, we divided our cases into two groups: early tremor onset (defined by tremor onset at the age of ≤40 years) and late tremor onset (tremor onset at the age of >40 years) (25). There were no differences between these two groups when we compared the percentage of times that each case reported worsening (64.3 ± 31.4 vs. 60.5 ± 36.3, *p* = 0.67).

**Table 3:** **Clinical correlates of self-reported worsening**.

	Correlation with percentage of times that cases reported worsening or mean ± SD [median] of percentage of times that cases reported worsening	*p*-Value
**Demographic variables**		
Age (years)	*r* = −0.01	0.90^a^
Gender		
Male	59.3 ± 35.2 [66.7]	0.40^b^
Female	63.8 ± 33.7 [71.4]	
Education (years)	*r* = −0.14	0.08^a^
Family history of ET		
Yes	67.1 ± 35.7 [75.0]	0.19^b^
No	60.4 ± 33.7 [66.7]	
**Tremor duration and severity**		
Tremor duration (years)	*r* = 0.003	0.97^a^
Age at tremor onset (years)	*r* = 0.003	0.97^a^
Total tremor score	*r* = 0.04	0.60^a^
Change in total spiral score	*r* = 0.08	0.35^a^
**Overall health and medications**		
CIRS score	*r* = 0.12	0.12^a^
Change in ethanol intake score	*r* = 0.03	0.69^a^
Medication change score	*r* = −0.12	0.12^a^
**Cognitive screen**		
MMSE score	*r* = 0.13	0.10^a^

*Unless indicated otherwise, all values are baseline values*.

*Data are mean ± SD [median] or Spearman’s rho*.

*CIRS, Cumulative Illness Rating Scale (range 0–42); MMSE, Folstein Mini Mental State Examination (range 0–30)*.

*^a^Spearman’s rho (*r*)*.

*^b^Mann–Whitney test*.

Second, ET cases were divided into quartiles based on the percentage of times they had indicated worsening of symptoms during follow-ups (Table 4). The analysis showed that the quartiles were similar in terms of basic demographic variables (baseline age, gender, education, family history, Table 4). There were no significant differences amongst the quartiles when we compared them by tremor duration, age at tremor onset, and total tremor score (Table 4). The quartiles did not differ by CIRS scores. The change in ethanol intake score differed marginally across quartiles but not in an ordinal manner. There was a marginally significant decrease in medication change score with increasing quartile; in other words, greater self-reported worsening was associated with greater use of tremor-reducing medication from baseline to follow-up. There was no progression of MMSE scores by quartile (Table 4).

**Table 4:** **Clinical correlates of reported worsening: quartile of worsening by demographic and clinical features**.

Quartile	First	Second	Third	Fourth	*p*-Value
*N*	41 (25.0)	42 (25.6)	37 (22.6)	44 (26.8)	
Percentage of time that cases reported worsening	12.0 ± 12.4 [11.1]	55.7 ± 10.1 [56.3]	79.9 ± 6.0 [80]	100 ± 0.0 [100]	
**Demographic variables**					
Age (years)	83.6 ± 6.5 [84.0]	83.4 ± 4.8 [84.0]	82.6 ± 5.4 [83.0]	83.7 ± 5.8 [83.5]	0.85^a^
Male gender	16 (39.0)	19 (45.2)	12 (32.4)	15 (34.1)	0.63^b^
Education (years)	15.5 ± 4.1 [16.0]	16.0 ± 3.6 [16.0]	14.4 ± 3.7 [14.0]	14.1 ± 4.7 [16.0]	0.30^c^
Family history of ET	10 (24.3)	8 (19.0)	9 (24.3)	15 (34.1)	0.45^b^
**Tremor duration and severity**					
Tremor duration (years)	36.7 ± 23.6 [35.0]	45.9 ± 23.7 [40.0]	41.2 ± 18.8 [35.0]	39.1 ± 23.0 [32.5]	0.21^c^
Age at tremor onset (years)	46.9 ± 22.0 [48.0]	37.5 ± 24.3 [42.5]	41.5 ± 21.1 [45.0]	44.5 ± 24.2 [52.5]	0.21^c^
Total tremor score	22.5 ± 7.0 [23.3]	25.0 ± 5.3 [26.3]	24.6 ± 6.6 [24.3]	23.3 ± 5.8 [23.5]	0.27^a^
Change in total spiral score	0.17 ± 2.8 [0.13]	−0.07 ± 2.7 [0.25]	0.13 ± 2.9 [0.75]	0.67 ± 2.4 [0.50]	0.80^c^
**Overall health and medications**					
CIRS score	9.63 ± 5.4 [9.0]	10.7 ± 5.5 [11.0]	9.76 ± 4.9 [10.0]	11.6 ± 5.3 [12.0]	0.26^c^
Change in ethanol intake score	−2.02 ± 4.9 [0.0]	−5.9 ± 19.7 [−1.0]	−1.64 ± 5.9 [0.0]	−3.3 ± 13.5 [0.0]	0.06^c^
Medication change score	0.37 ± 0.9 [0.0]	0.21 ± 1.0 [0.0]	0.11 ± 0.8 [0.0]	−0.07 ± 1.3 [0.0]	0.27^c^
					0.05^d^
**Cognitive screen**					
MMSE score	26.4 ± 2.7 [27.0]	27.6 ± 1.9 [28.0]	27.1 ± 2.3 [27.0]	27.2 ± 4.4 [28.0]	0.12^c^

*Unless indicated otherwise, all values are baseline values*.

*Data are mean ± SD [median] or number (percentage)*.

*CIRS, Cumulative Illness Rating Scale (range 0–42); MMSE, Folstein Mini Mental State Examination (range 0–30)*.

*^a^ANOVA*.

*^b^Chi-square test*.

*^c^Kruskal–Wallis test*.

*^d^Jonckheere–Terpstra test*.

### Correlate of Self-Reported Worsening and Change in Spiral Scores

We also assessed the correlation between subjective worsening and an objective measure of worsening (i.e., change in spiral scores). As described above, two of the authors rated all of the spirals obtained at baseline and at the most recent follow-up evaluations using the Bain and Findley 10-point scale (20) and calculated a change in total spiral score for each case. We then explored the correlation between the change in total spiral score and the percentage of times our cases reported worsening. There was no correlation (Spearman’s *r* = 0.08, *p* = 0.35).

In order to consider the potential impact of ET treatments on these analyses, we performed sensitivity analyses. First, we restricted the analysis to those cases (*N* = 32) who had never taken tremor-reducing medications, and this produced similar results (Spearman’s *r* = 0.19, *p* = 0.37, i.e., no correlation between percentage of times our cases reported worsening and change in total spiral score). We repeated this analysis with cases (*N* = 116) who were always on at least one tremor-reducing medication. Again, there was no correlation between self-reported worsening and change in total spiral score (Spearman’s *r* = 0.07, *p* = 0.48).

## Discussion

To our knowledge, this is the first prospective longitudinal study to examine the progression of symptoms from the perspective of the ET patient. On average, these ET cases were followed for 4 years, and each case completed an average of seven follow-up assessments. During this time, most cases [145 (88.4%)] reported a worsening of their symptoms at least once. A majority [104 cases (63.4%)] reported worsening during at least 50% of the follow-ups. Only a very small group [19 (11.6%)] did not report any worsening. A significant minority of cases [44 (26.8%)] reported worsening at every single follow-up, indicating that they felt they were inexorably getting worse and worse and worse with time.

That there is so much self-reported worsening in ET argues against the notion that this is a static and benign condition. It suggests that patients experience it as a condition that worsens consistently and regularly.

Two of the authors rated the spirals obtained at baseline and at the most recent follow-up evaluation using the Bain and Findley 10-point scale (20) and calculated a change in total spiral score for each case. We did not find a correlation between the percentage of times our cases reported worsening and the change in total spiral score. This suggests that there is a subjective component to self-reported worsening that may not be borne out by independent objective evaluations. Another possibility is that the change noticed by the patients may be objectively small. A previous study that examined the change in spiral scores in ET patients using this same scale over time determined that scores increased at an average rate of 0.12 ± 0.23 points per year. The rate of increase was so small that the change in scores was apparent to a blinded neurologist only in cases that had been followed for at least 5 years (26). Given that the mean length of follow-up in our study was a little less than 4 years, a longer time span or a more precise rating scale might be needed to better assess this association. Additionally, since tremor in ET is known to vary from moment to moment (27), a different tool that provides continuous monitoring (vs. the snapshot in time that spiral drawing offers) may be required to overcome this intra-subject variability. Automated measures of tremor, such as wearable equipment, may be suited to capture the objective worsening of symptoms.

We looked at the association of self-reported worsening of symptoms and changes in ethanol consumption. In the past, the idea that ET patients self-medicate with ethanol leading to a higher consumption amongst patients with more severe symptoms had gained some traction. Although a marginal difference in change in ethanol consumption was apparent when cases were divided into quartiles based on the percentage of times they reported worsening, the mean scores in each quartile did not differ in an ordinal manner. Furthermore, we did not find a correlation between change in ethanol consumption and self-reported worsening in our cohort. The absence of a correlation argues against the idea that ET patients are using ethanol to self-medicate and supports recently published data on this subject (28).

The results from this study can provide clinicians with useful information to address their patients’ inquiries about the prognosis of their disease. As mentioned above, it appears that a significant portion of ET patients will experience what they feel as a progressive worsening of symptoms despite their physicians’ best efforts. Given this information, clinicians could benefit from setting appropriate expectations of treatment effects.

This study should be considered in the context of certain limitations. First, these cases were highly selected because many of them were ascertained through a disease-specific organization and because they self-referred to the ETCBR as future brain donors. These cases may not be representative of the general ET patient population as they may suffer from more severe disease. Therefore, a community-based study would better estimate the progression of symptoms in the community. Second, although we tried to assess the impact of changes of individual medications throughout follow-up, we did not have access to information on changes of dosages. To explore the possibility that changes of dosages may have affected our results, we repeated our analyses restricting the cases to those who were never on any medications. Self-reported worsening was similar to that which we reported in our main analysis. Finally, future longitudinal studies exploring self-reported progression of symptoms could benefit from the inclusion of additional health-related quality of life instruments, such as the Quality of Life in Essential Tremor (QUEST) questionnaire (29) or the EuroQol 5-Dimension questionnaire (30).

In summary, when followed longitudinally at regular intervals, most ET cases (88.4%) reported a worsening of their symptoms at least once, 63.4% reported worsening during at least 50% of the follow-ups and one in four cases reported worsening at every single follow-up assessment, indicating that they felt they were inexorably getting worse and worse and worse with time.

## Author Contributions

JG: research project: organization and execution; statistical analysis: design, execution, and review and critique; and manuscript preparation: writing of the first draft and review and critique. JP: research project: execution; statistical analysis: design, execution, and review and critique; and manuscript preparation: review and critique. OB: research project: execution; manuscript preparation: review and critique. EL: research project: conception; statistical analysis: design and review and critique; and manuscript preparation: writing of the first draft and review and critique.

## Conflict of Interest Statement

The authors declare that the research was conducted in the absence of any commercial or financial relationships that could be construed as a potential conflict of interest.
